# Legacy Effect of Delayed Blood Pressure-Lowering Pharmacotherapy in Middle-Aged Individuals Stratified by Absolute Cardiovascular Disease Risk: Protocol for a Systematic Review

**DOI:** 10.2196/resprot.8362

**Published:** 2017-09-01

**Authors:** Chau Le Bao Ho, Sharon Sanders, Jenny Doust, Monique Breslin, Christopher M Reid, Mark Raymond Nelson

**Affiliations:** ^1^ Menzies Institute for Medical Research University of Tasmania Hobart, TAS Australia; ^2^ Centre for Research in Evidence-Based Practice Bond University Gold Coast, QLD Australia; ^3^ School of Public Health Curtin University Perth, WA Australia; ^4^ Centre of Cardiovascular Research & Education in Therapeutics, School of Public Health and Preventive Medicine Monash University Melbourne, VIC Australia

**Keywords:** legacy effect, high blood pressure, cardiovascular disease

## Abstract

**Background:**

Many national and international guidelines recommend that the initiation of blood pressure (BP)-lowering drug treatment for the primary prevention of cardiovascular disease (CVD) should no longer be based on BP level alone, but on absolute cardiovascular risk. While BP-lowering drug treatment is beneficial in high-risk individuals at any level of elevated BP, clinicians are concerned about legacy effects on patients with low-to-moderate risk and mildly elevated BP who remain “untreated”.

**Objective:**

We aim to investigate the legacy effect of delayed BP-lowering pharmacotherapy in middle-aged individuals (45-65 years) with mildly elevated BP (systolic BP 140-159 mmHg and/or diastolic BP 90-99 mmHg) stratified by absolute risk for primary prevention of CVD, but particularly in the low-risk (<10% five-year absolute risk) group.

**Methods:**

Randomized trials of BP-lowering therapy versus placebo or pretreated subjects in active comparator studies with posttrial follow-up will be identified using a 2-step process. First, randomized trials of BP-lowering therapy will be identified by (1) retrieving the references of trials included in published systematic reviews of BP-lowering therapy, (2) retrieving studies published by the Blood Pressure Lowering Treatment Trialists’ Collaboration (BPLTTC), and (3) checking studies referenced in the 1993 World Health Organization/International Society of Hypertension meeting memorandum on BP management. Posttrial follow-up studies will then be identified by forward citation searching the randomized trials identified in step 1 through Web of Science. The search will include randomized controlled trials with at least 1-year in-trial period and a posttrial follow-up phase. Age is the major determinant of absolute cardiovascular risk, so the participants in our review will be restricted to middle-aged adults who are more likely to have a lower cardiovascular risk profile. The primary outcome will be all-cause mortality. Secondary outcomes will include cardiovascular mortality, fatal stroke, fatal myocardial infarction, and death due to heart failure.

**Results:**

The searches for existing systematic reviews and BPLTTC studies were piloted and modified. The study is expected to be completed before June 2018.

**Conclusions:**

The findings of this study will contribute to the body of knowledge concerning the beneficial, neutral, or harmful effects of delayed BP-lowering drug treatment on the primary prevention of CVD in patients with mildly elevated BP and low-to-moderate CVD risk.

**Trial Registration:**

PROSPERO International Prospective Register of Systematic Reviews: CRD42017058414; https://www.crd.york.ac.uk/PROSPERO/display_record.asp?ID=CRD42017058414 (Archived by WebCite® at http://www.webcitation.org/6t6sa8O2Q)

## Introduction

Despite improvements in the management of cardiovascular disease (CVD) over the past five decades, it remains the leading cause of death and disability in the world [1]. CVD was responsible for approximately 17.5 million deaths worldwide in 2012 [1]. In updated guidelines for the primary prevention of CVD from Australasia [2,3], the United Kingdom [4], and Europe [5], blood pressure (BP)-lowering pharmacotherapy is indicated by absolute CVD risk, not BP level alone. In contrast, the US guideline (the Eighth Joint National Committee) [6] is still heavily focused on BP level and age, despite the fact that BP-lowering therapy is beneficial for the reduction of CVD mortality and morbidity at sufficiently high CVD absolute risk, regardless of the level of BP elevation [7]. The use of BP-lowering drug treatment in high-risk settings has achieved consensus in Australasian [2,3], European [5], and the US guidelines [6]. In low-risk individuals, BP-lowering drugs are not recommended by guidelines in Australia [2], New Zealand [3] or the United Kingdom [4], unless BP exceeds a level of 160/100 mmHg, whereas the European [5] and US [6] guidelines recommended an early initiation at a BP level of 140/90 mmHg. However, both approaches raised many concerns from clinicians and a gap still exists between guidelines and clinical practice [8].

An international expert consultation was recently performed to solve the controversy of whether adults with grade 1 hypertension (<140/90 mmHg) and low-to-moderate CVD risk should be treated by drug therapy [9]. Morales-Salinas et al [9] recommended an early initiation of BP-lowering pharmacotherapy primarily from the results of the Heart Outcomes Prevention Evaluation (HOPE-3) trial [10] and a meta-analysis by Thomopoulos et al [11] for adults with grade 1 hypertension and moderate CVD risk; however, the two studies were likely to include a number of high-risk participants. In the HOPE-3 trial [10], participants with an INTERHEART risk score higher than 16 (a value of 16 or higher indicates a high CVD risk) accounted for 32.5% of the total sample [12]. In the meta-analysis by Thomopoulos et al [11], the CVD risk was calculated by CVD death rate in the control group, while the CVD risk score used in most guidelines is for fatal and nonfatal CVD events. Thus, the benefits of BP-lowering pharmacotherapy in low-to-moderate-risk individuals remain unclear, as opposed to the benefits achieved by treating high risk individuals. Most clinicians use BP-lowering pharmacotherapy based on BP criteria alone, due to the perceived potential risk of irreversible target organ damage (the “legacy effect”) for delayed therapy [5]. Studies that would help us to answer this question include those that have extended follow-up in the posttrial period. Such studies include the Systolic Hypertension in the Elderly Program trial [13] of approximately 22 years, the Hypertension Detection and Follow-Up Program [14] of 8.3 years, and the second Australian National Blood Pressure study [15] of 10.6 years. Participants in these studies are still likely to be at high baseline risk of CVD due to the advanced age and diabetic status in the inclusion criteria of the trials [13-15]. Hence, the concern of legacy effects on low-to-moderate-risk individuals has not been addressed. Age is the most important determinant of adverse cardiovascular risk, so the participants in our review are restricted to middle-aged adults who are more likely to have a broader cardiovascular risk profile. Therefore, in this systematic review and meta-analysis, we will investigate the effects of BP-lowering drug treatments in middle-aged individuals with mildly elevated BP, stratified by absolute CVD risk.

## Methods

### Review Objectives

#### Aim 1

We will conduct a systematic review and meta-analysis of published and unpublished studies of randomized placebo control trials with a posttrial follow-up phase that included middle-aged participants without overt CVD, and examine these studies for CVD mortality and all-cause mortality.

#### Aim 2

We will conduct a subgroup analysis (where possible) of participants in these trials classified as low-, moderate-, and high-absolute CVD risk by the Framingham Risk Score (FRS) used in the Australia guideline [2], or the risk calculator used by the Blood Pressure Lowering Treatment Trialists’ Collaboration (BPLTTC) [16] which uses routine clinical information if information on cholesterol levels is not available for fatal and nonfatal CVD events and all-cause mortality. We will conduct an individual patient data meta-analysis, if data are available.

### Primary Null Hypothesis

There will be no significant difference in CVD mortality or all-cause mortality between patients who have drug therapy initiated earlier (active treatment arm) versus delayed or not initiated (control arm) in individuals at low-absolute CVD risk.

### Secondary Hypotheses

#### Hypothesis 1

There will be no significant difference in CVD mortality or all-cause mortality between patients who have drug therapy initiated earlier (active treatment arm) versus delayed or not initiated (control arm) in individuals at moderate-absolute CVD risk.

#### Hypothesis 2

There will be no significant difference in CVD mortality or all-cause mortality between patients who have drug therapy initiated earlier (active treatment arm) versus delayed or not initiated (control arm) in individuals at high-absolute CVD risk.

#### Hypothesis 3

In-trial CVD events (fatal and nonfatal) will be incremental by risk classification estimated by FRS or equivalent risk calculated at baseline.

### Criteria for Considering Studies in the Review

#### Population

The study will include men and nonpregnant women from 45 to 65 years of age. At least 80% of participants from each trial must have had mildly elevated BP at baseline, defined as a systolic BP of 140- 159 mmHg and/or diastolic BP 90- 99 mmHg. Furthermore, all included participants must not have exhibited any history of CVD at baseline: myocardial infarction, angina pectoris, coronary bypass surgery, coronary angioplasty, stroke, transient ischaemic attack, carotid endarterectomy, surgery for peripheral vascular disease, intermittent claudication or renal failure (creatinine >1.5 times the upper limit of normal). If trials included participants different than those of interest (eg, secondary prevention, moderately-elevated or highly-elevated BP), we will attempt to access individual patient data and subsequently select participants that meet specific criteria.

#### Intervention

The study will focus on all types of BP-lowering drugs, except for some types that have limited clinical use due to the risk of side effects and availability (eg, ganglion blockers, reserpine, rauwolfia).

#### Comparison

The study will compare the effects of BP-lowering drug treatments in active treatment groups versus control treatment groups. However, if comparative trials with two active comparators had an extended posttrial follow-up phase and individual data are available, we will perform a legacy effect analysis per Nelson et al [15]. We will reclassify participants into *previous treatment* (early treatment) groups and *treatment naïve* (delayed treatment) groups. The *previous treatment* group will include participants who were on BP-lowering drug treatments at trial registration and then went on a specific drug withdrawal program. The *treatment naïve* group will include those who were not on any treatments at trial registration.

#### Outcomes

Primary outcomes will include all-cause mortality in both randomization and follow-up periods. Secondary outcomes will include CVD mortality (defined as deaths due to stroke, myocardial infarction, and heart failure), fatal stroke, fatal myocardial infarction, and fatal heart failure. Nonfatal CVD events will be included if the measurements of outcomes are similar between trials. Vital status in posttrial periods must be assessed by national death databases or equivalent records.

#### Study Design

Randomized controlled trials with at least 1-year in-trial period and a posttrial follow-up phase.

#### Language

No restriction (English and non-English studies).

#### Publication Type

Published and unpublished studies reported in peer-reviewed journals, reports, conference abstracts, and theses.

### Search Methods for Identification of Studies

Randomized trials of BP-lowering therapies versus placebo or active comparator with posttrial follow-up periods will be identified using a 2-step process. First, randomized trials of BP-lowering therapy will be identified by (1) retrieving the references of trials included in published systematic reviews of BP-lowering therapy, (2) retrieving studies published by the BPLTTC, and (3) checking studies referenced in the 1993 WHO/ISH (World Health Organization/International Society of Hypertension) meeting memorandum on BP management [17]. To identify existing systematic reviews, we will search Medline Ovid using a combination of Medical Subject Headings and text word terms for BP-lowering regimes and high BP with a systematic review filter (see Multimedia Appendix 1). Web of Science will be used to retrieve the references of studies cited by the systematic reviews, and these will be exported to an Endnote file. To identify studies from the BPLTTC, a text word search in the title, abstract, and author fields will be conducted in Ovid Medline and the retrieved references will be exported to the Endnote file. Web of Science will be used to retrieve the references of studies cited in the WHO/ISH meeting memorandum and these will be exported to the Endnote file. After removing duplications, the Endnote file will be screened to identify randomized trials of BP-lowering therapies versus placebo or active comparators. In the second step of the search, posttrial follow-up studies will be identified by forward citation searching the randomized trials identified in step 1. Web of Science will be used for forward citation searches of each of the original trials, with the citations exported to another Endnote file. After the removal of duplications in the Endnote file, the file will be searched using terms related to extended follow-up (see Multimedia Appendix 2). The resulting titles and abstracts will be screened independently by two reviewers using the review eligibility criteria.

### Study Selection

First, two independent reviewers will screen a small sample of papers found in the search to revise any unclear or inappropriate inclusion criteria. In the full selection process, two reviewers will independently scan the results of the search and determine the eligibility of the studies. In the initial screening of titles and abstracts, the studies will be included if they meet the inclusion criteria or they do not have enough information for exclusion. Rejected citations will be recorded and classified as irrelevant studies. All potentially relevant articles will be screened through full text for a final decision. If a paper does not have sufficient information to assess eligibility, we will attempt to contact the authors; the paper will be classified as a *potentially relevant article* and checked in sensitivity analyses if authors do not reply after one month. If we identify trials that meet our inclusion criteria but lack data on the posttrial follow-up period, we will run a forward citation search from those studies. If a study has multiple citations, we will report separate citations but analyze these reports as a single study. We will also liaise with the BPLTTC for any individual patient data from trials meeting our inclusion criteria.

### Data Extraction and Quality Assessment

Data extraction forms (detailed in Textbox 1) and quality assessment forms will be piloted on a small group of studies. Two reviewers will independently perform data extraction and quality assessment in the prespecified form. If any disagreements arise, the reviewers will discuss consensus or consult with the third reviewer. A report of correction or amendments to the prespecified form will be recorded.

**Information required for data extraction.**

*General information:* reviewer performing data extraction, date of data extraction, and identification features of the study (eg, record number, authors, article title, type of publication, country of origin, the source of funding)

*Study characteristics:* aims of the study, study design, study inclusion and exclusion criteria, recruitment procedures (details of randomization, blinding), and unit of allocation (participant, GP practice)

*Participant characteristics:* baseline characteristics (age, gender, ethnicity, socioeconomic status, comorbidities, systolic BP, diastolic BP, weight, height, smoking status, serum total cholesterol, serum creatinine level), and the number of participants in active treatment group and control group

*Intervention and setting*: type and dose of BP-lowering regimen

Outcome data:

- For each outcome: whether reported, definition, length of follow-up, number of events, number of participants in each event, odds ratio, risk ratio, and hazard ratio
- For both intervention groups: number of participants enrolled; number of participants included in analysis; and number of withdrawals, exclusions, and lost to follow-up

*Type of analysis used in the study:* intention to treat or per protocol

### Quality Assessment

The risk of bias will be assessed by two reviewers following the Cochrane Risk of bias tool [18] which includes the following criteria: random sequence generation (selection bias); allocation concealment (selection bias); blinding of participants, personnel, and outcome (performance and detection bias); and incomplete outcome data (attrition bias). The bias will be assessed as unclear, low-risk, or high-risk. Publication bias will be judged by observing the asymmetry of funnel plots; if they are asymmetric, contour-enhanced funnel plots will then be analyzed to examine whether publication bias alone caused the asymmetry. We will also use Egger’s meta-regression model to assess the relationship between the observed effect sizes and the size of studies [19].

### Data Synthesis and Analysis

After pooling all eligible studies, we will design a fixed-effect model and assess the heterogeneity by visually inspecting the forest plots, Chi-squared tests, and I^2^tests. Statistical heterogeneity will be recorded when the studies’ confidence intervals exhibit poor overlap, the P-value of the test of heterogeneity is 0.1 or lower, or the I^2^value is 0.5 or greater. In these cases, we will also perform an analysis using a random-effects model. All trial endpoints will be treated as dichotomous variables and grouped by time from randomization. In the fixed-effect model, the Mantel-Haenszel method model will be used to combine risk ratios of each outcome [20]. We will conduct a subgroup analysis in which available risk calculators will be used to stratify participants by the baseline absolute CVD risk for fatal and nonfatal CVD events. In a sensitivity analysis, each study will be removed (one at a time) to assess the impact of each study on the pooled outcomes. The Cochrane software (Revman) [21] will be used for meta-analysis, selective reporting, and other sources of bias.

### Ethics and Dissemination

This systematic review will analyze nonidentifiable data; thus, a formal ethics approval is unlikely to be crucial. The study protocol was registered with the International Prospective Register of Systematic Review (PROSPERO) with the reference number CRD42017058414. The current study will contribute a chapter of a PhD thesis (CH).

## Results

We are currently in the process of developing the search strategy. The search in Medline via Ovid has been piloted and modified. The analysis is expected to complete before June 2018.

## Discussion

Given the strong beliefs held by many clinicians that early treatment of elevated BP is necessary to prevent CVD events, it is not possible to conduct a randomized controlled trial of early versus late treatment at present. This is particularly true for patients with mildly elevated BP and low CVD risk, as studies would require a large sample size of participants or a long follow-up period because approximately 10% of CVD events are expected to occur within 10 years. In addition, clinicians are questioning the real benefits, adverse effects, and medical costs of the life-long intervention of BP-lowering drug treatment. The findings of this study will contribute to the body of knowledge concerning the beneficial, neutral, or harmful effects of delayed BP-lowering drug treatment in patients with mildly elevated BP and low-to-moderate CVD risk.

### Limitations

Due to the changes in definitions of CVD and diagnostic methods used over time, we predict that it will be difficult to combine these outcomes in a meta-analysis. This issue inherently generates bias in selection, detection, attrition, and reporting.

## Appendix

Multimedia Appendix 1 Search strategy developed for Medline via Ovid to identify existing systematics review.

Multimedia Appendix 2 Search terms related to extended follow-up.

## Abbreviations

BP: blood pressure
BPLTTC: Blood Pressure Lowering Treatment Trialists’ Collaboration
CVD: cardiovascular disease
FRS: Framingham Risk Score
HOPE-3: Heart Outcomes Prevention Evaluation trial
WHO/ISH: World Health Organization/International Society of Hypertension
