# Supplementary material for: Legacy Effect of Delayed Blood Pressure-Lowering Pharmacotherapy in Middle-Aged Individuals Stratified by Absolute Cardiovascular Disease Risk: Protocol for a Systematic Review
Source: JMIR Res Protoc. 2017 Sep 1;6(9):e177. doi: 10.2196/resprot.8362 (PMC5600968; doi:10.2196/resprot.8362)
Supplement: Multimedia Appendix 1 [file resprot_v6i9e177_app1.pdf]

Multimedia Appendix 1. Search strategy developed for Medline via Ovid to identify existing systematics review.

Concept 1: BP lowering regimens

1 exp antihypertensive agents/

2 (antihypertensive$ adj (agent$ or drug)).tw.

3 exp thiazides/

4 (chlorothiazide or benzothiadiazine or bendroflumethiazide or cyclopenthiazide or metolazone or xipamide or hydrochlorothiazide or hydroflumethiazide or methyclothiazide or polythiazide or trichlormethiazide or thiazide?).tw.

5 (chlorthalidone or chlortalidone or phthalamudine or chlorphthalidolone or oxodolin or thalitone or hygroton or indapamide or metindamide).tw.

6 ((loop or ceiling) adj diuretic?).tw.

7 (bumetanide or furosemide or torasemide).tw.

8 exp sodium potassium chloride symporter inhibitors/

9 (eplerenone or amiloride or spironolactone or triamterene).tw.

10 or/1-9

11 exp angiotensin-converting enzyme inhibitors/

12 ((angiotensin$ or kininase ii or dipeptidyl$) adj3 (convert$ or enzyme or inhibit$ or recept$)).tw.

13 (ace adj3 inhibit$).tw.

14 acei.tw.

15 exp enalapril/

16 (alacepril or altiopril or benazepril or captopril or ceronapril or cilazapril or delapril or enalapril or fosinopril or idapril or imidapril or lisinopril or moexipril or moveltipril or pentopril or perindopril or quinapril or ramipril or spirapril or temocapril or trandolapril or zofenopril or teprotide).tw.

17 or/11-16

18 exp Angiotensin II Type 1 Receptor Blockers/

19 (angiotensin$ adj4 receptor$ adj3 (antagon$ or block$)).tw.

20 exp losartan/

21 (KT3-671 or candesartan or eprosartan or irbesartan or losartan or olmesartan or tasosartan or telmisartan or valsartan or azilsartan or saralasin).tw.

22 or/18-21

23 exp calcium channel blockers/

24 (calcium adj2 (inhibit$ or antagonist? or block$)).tw.

25 (calcium channel block$ or amlodipine or amrinone or bencyclane or bepridil or cinnarizine or conotoxins or diltiazem or felodipine or fendiline or flunarizine or gallopamil or isradipine or lidoflazine or lacidipine or lercanidipine or magnesium sulfate or mibefradil or nicardipine or nifedipine or nimodipine or nisoldipine or nitrendipine or perhexiline or prenylamine or verapamil or omega-agatoxin iva or omega-conotoxin gvia or omega-conotoxins).tw.

26 or/23-25

27 exp adrenergic beta-antagonists/

28 adrenergic beta antagonist?.tw.

29 (beta adj2 (antagonist? or receptor? or adrenergic? block$)).tw.

30 (acebutolol or adimolol or afurolol or alprenolol or amosulalol or arotinolol or atenolol or befunolol or betaxolol or bevantolol or bisoprolol or bopindolol or bornaprolol or brefonalol or bucindolol or bucumolol or bufetolol or bufuralol or bunitrolol or bunolol or bupranolol or butofilolol or butoxamine or carazolol or carteolol or carvedilol or celiprolol or cetamolol or chlortalidone cloranolol or cyanoiodopindolol or cyanopindolol or deacetylmetipranolol or diacetolol or dihydroalprenolol or dilevalol or epanolol or esmolol or exaprolol or falintolol or flestolol or flusoxolol or hydroxybenzylpinodolol or hydroxycarteolol or hydroxymetoprolol or indenolol or iodocyanopindolol or iodopindolol or iprocrolol or isoxaprolol or labetalol or landiolol or levobunolol or levomoprolol or medroxalol or mepindolol or methylthiopropranolol or metipranolol or metoprolol or moprolol or nadolol or oxprenolol or penbutolol or pindolol or nadolol or nebivolol or nifenalol or nipradilol or oxprenolol or pafenolol or pamatolol or penbutolol or pindolol or practolol or primidolol or prizidilol or procinolol or pronetalol or propranolol or proxodolol or ridazolol or salcardolol or soquinolol or sotalol or spirendolol or talinolol or tertatolol or tienoxolol or tilisolol or timolol or tolamolol or toliprolol or tribendilol or xibenolol).tw.

31 or/27-30

32 exp adrenergic alpha antagonists/

33 (adrenergic adj2 (alpha or antagonist?)).tw.

34 ((adrenergic or alpha or receptor?) adj2 block$).tw.

35 (alfuzosin or bunazosin or doxazosin or metazosin or indoramin or neldazosin or prazosin or silodosin or tamsulosin or terazosin or tiodazosin or trimazosin).tw.

36 or/32-35

37 exp hydralazine/

38 (minoxidil or methyldopa or clonidine or moxonidine or aliskiren or enalkire or remikiren or imidazole$ or methyldopa or dopamet or alphamethyldopa or clonidine).tw.

39 or/37-38

40 10 or 17 or 22 or 26 or 31 or 36 or 39

Concept 2: High blood pressure

41 hypertension/

42 hypertens$.tw.

43 ((high or elevat$ or rais$) adj2 blood pressure).tw.

44 or/41-43

Concept 3: Study design

45 meta analysis.pt.

46 meta analysis.ti,ab.

47 meta analysis/

48 search*.tw.

49 systematic review.tw.

50 or/ 45-49

Search hit

51 40 and 44 and 50
