# Supplementary material for: Legacy Effect of Delayed Blood Pressure-Lowering Pharmacotherapy in Middle-Aged Individuals Stratified by Absolute Cardiovascular Disease Risk: Protocol for a Systematic Review
Source: JMIR Res Protoc. 2017 Sep 1;6(9):e177. doi: 10.2196/resprot.8362 (PMC5600968; doi:10.2196/resprot.8362)
Supplement: Multimedia Appendix 2 [file resprot_v6i9e177_app2.pdf]

Multimedia Appendix 2. Search terms related to extended follow-up

1. Post-trial

2. Posttrial

3. Follow-up

4. Followup

5. Extension

6. Long-term

7. Longterm

8. Extended observation

9. Extended study

10. Legacy
